# Mice Deficient in Ribosomal Protein S6 Phosphorylation Suffer from Muscle Weakness that Reflects a Growth Defect and Energy Deficit

**DOI:** 10.1371/journal.pone.0005618

**Published:** 2009-05-19

**Authors:** Igor Ruvinsky, Maximiliano Katz, Avigail Dreazen, Yuval Gielchinsky, Ann Saada, Nanette Freedman, Eyal Mishani, Gabriel Zimmerman, Judith Kasir, Oded Meyuhas

**Affiliations:** 1 Department of Biochemistry and Molecular Biology, IMRIC, Hebrew University-Hadassah Medical School, Jerusalem, Israel; 2 Department of Obstetrics and Gynecology, Hadassah Medical Center, Jerusalem, Israel; 3 Metabolic Disease Unit, Hadassah Medical Center, Jerusalem, Israel; 4 Department of Medical Biophysics and Nuclear Medicine, Hadassah Medical Center, Jerusalem, Israel; 5 Department of Biological Chemistry, Institute of Life Sciences, The Hebrew University, Jerusalem, Israel; Universidade Federal do Rio de Janeiro (UFRJ), Instituto de Biofísica da UFRJ, Brazil

## Abstract

**Background:**

Mice, whose ribosomal protein S6 cannot be phosphorylated due to replacement of all five phosphorylatable serine residues by alanines (rpS6^P−/−^), are viable and fertile. However, phenotypic characterization of these mice and embryo fibroblasts derived from them, has established the role of these modifications in the regulation of the size of several cell types, as well as pancreatic β-cell function and glucose homeostasis. A relatively passive behavior of these mice has raised the possibility that they suffer from muscle weakness, which has, indeed, been confirmed by a variety of physical performance tests.

**Methodology/Principal Findings:**

A large variety of experimental methodologies, including morphometric measurements of histological preparations, high throughput proteomic analysis, positron emission tomography (PET) and numerous biochemical assays, were used in an attempt to establish the mechanism underlying the relative weakness of rpS6^P−/−^ muscles. Collectively, these experiments have demonstrated that the physical inferiority appears to result from two defects: a) a decrease in total muscle mass that reflects impaired growth, rather than aberrant differentiation of myofibers, as well as a diminished abundance of contractile proteins; and b) a reduced content of ATP and phosphocreatine, two readily available energy sources. The abundance of three mitochondrial proteins has been shown to diminish in the knockin mouse. However, the apparent energy deficiency in this genotype does not result from a lower mitochondrial mass or compromised activity of enzymes of the oxidative phosphorylation, nor does it reflect a decline in insulin-dependent glucose uptake, or diminution in storage of glycogen or triacylglycerol (TG) in the muscle.

**Conclusions/Significance:**

This study establishes rpS6 phosphorylation as a determinant of muscle strength through its role in regulation of myofiber growth and energy content. Interestingly, a similar role has been assigned for ribosomal protein S6 kinase 1, even though it regulates myoblast growth in an rpS6 phosphorylation-independent fashion.

## Introduction

The phosphorylation of ribosomal protein S6 (rpS6) was first demonstrated in regenerating rat liver [1] and subsequently in response to numerous physiological, pathological and pharmacological stimuli ([2] and references therein). The five clustered phosphorylatable serine residues in rpS6 are located at the carboxy terminus (S235, S236, S240, S244 and S247) and are evolutionarily conserved in higher eukaryotes [3]. Mammalian cells contain two forms of rpS6 kinase, S6K1 and S6K2 [4]. S6K1^−/−^ mice are significantly smaller at birth, due to a proportional decrease in the size of all organs [5]. A smaller cell size in these mice was reported for pancreatic β-cells [6] and myoblasts [7]. In contrast, the birth weight of S6K2^−/−^ mice, as well as the size of their myoblasts, are similar to those of wild type mice [6], [7]. The embryonic and postnatal growth, like the size of myoblasts of the double knockout mice, S6K1^−/−^/S6K2^−/−^, are comparable with those of S6K1^−/−^ mice [7], [8], further underscoring the dominant role of S6K1 in cell size regulation. However, unlike the individual deficiency of each of these genes, the combined deletion of both S6Ks is associated with a profound decrease in viability [8].

AMPK is the downstream component of a pathway that acts as a sensor of cellular energy charge by monitoring AMP∶ATP ratio. Once activated, it switches on ATP-yielding processes, like glucose uptake, glycolysis and mitochondrial biogenesis, while switching off ATP-consuming processes, such as lipogenesis and glycogen synthesis [reviewed in [9]]. The small size of S6K-deficient muscle and myoblasts seems to be mediated by upregulation of AMP-activated kinase (AMPK) in response to an increased AMP∶ATP ratio in the mutant muscle [10]. Moreover, the increased content of mitochondria and the reduced level of triacylglycerol in this muscle are consistent with the apparent elevated AMPK activity [11], [12]. Accordingly, downregulation of AMPK protects S6K-deficient myotubes or myofibers from size decrease, suggesting that AMPK activity negatively contributes to the growth control of muscle cells [10]. However, given the multiplicity of S6K targets [3], the role of rpS6 phosphorylation in mediating cell size control is not self-evident. Furthermore, phosphorylation of rpS6 at S235 and S236 can still be detected, albeit at a much lower level, in S6K1^−/−^/S6K2^−/−^ cells [8], which correlates with a recent report implicating another ribosomal protein S6 kinase (RSK) in phosphorylation of rpS6 exclusively at S235 and S236 in response to various mitogenic signals [13].

The physiological role of rpS6 phosphorylation has just recently started being disclosed through a knockin mouse (rpS6^P−/−^), in which all five phosphorylatable serine residues in rpS6 were substituted by alanines [14]. These mice are viable, fertile and do not show a shorter life span, yet mouse embryo fibroblasts (MEFs) derived from this genotype are significantly smaller than rpS6^P+/+^ MEFs, and divide faster. This small size phenotype reflects a growth defect, rather than being a byproduct of their faster cell division and it is not confined to embryonal cells, since it also selectively characterizes pancreatic β-cells in adult rpS6^P−/−^ mice. The knockin mouse suffers from diminished level of pancreatic insulin, hypoinsulinemia and impaired glucose tolerance, as do S6K1^−/−^ mice [6], [14].

In this report we describe our attempts to elucidate the mechanism underlying the apparent reduced muscle strength in rpS6^P−/−^ mice. Our results indicate that myotubes of skeletal muscles in the knockin mouse have a smaller cross sectional area (CSA) due to a defect in myotubes growth. The resulting reduction in total muscle mass, together with a decrease in the abundance of contractile proteins, as well as the diminution in levels of ATP and creatine phosphate (PCr) provide an explanation for the impaired muscle function.

## Results

### rpS6 phosphorylation is required for maintaining muscle strength

**Figure 1:**
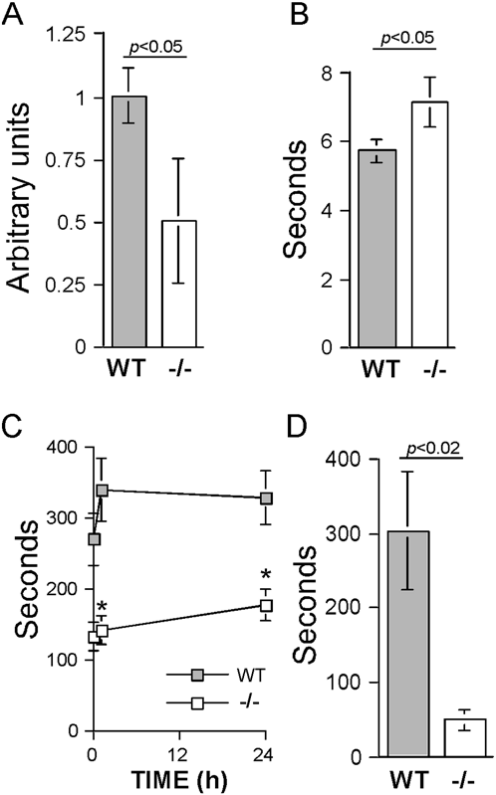
Muscle strength is impaired in rpS6^P−/−^ mice. (A to C) Age-matched (4 and 7 month) male mice (n = 13 for WT and 12 for rpS6^P−/−^ mice) were subjected to screening, according to the SHIRPA behavioral protocol (see details in “Experimental Procedures”). (A) Grip strength. Mice were allowed to grip a grid and a gentle horizontal backwards pull through their tail was applied. Higher scores indicate greater grip strength. An unbiased observer, blinded to the genotype, performed the experiment in a blind fashion; (B) Wire maneuver. Results represent time in seconds required for a mouse that is hung from a wire with its forearms to elevate its hind limbs and grip the wire. (C) Rota-rod performance. Mice were placed on a moving cylinder, which was gradually accelerated from an initial speed of 4 rpm to a maximum of 40 rpm. Latency to fall from the rota-rod is presented in seconds. Motor performance was measured in three 10 min sessions (time 0, 1 h and 24 h). In each trial, the time in seconds until falling off was recorded. *P<0.0005 for each trial versus rpS6^P+/+^ mice. (D) Endurance test. 5 rpS6^p+/+^ and 4 S6^P−/−^ age-matched (7–9 weeks) male mice were allowed to run on the treadmill set with a slope of 12.5 degree and a speed of 20 m/min. The results represent the total running time with two attempts to pause. Results of all experiments are presented as average±SEM, and the absence of SEM bars for some measurements simply reflects a value close to zero that is graphically invisible.

During routine handling of adult mice (older than 6 weeks) our attention was drawn to the diminished force displayed by rpS6^P−/−^ mice, relative to that of rpS6^P+/+^ mice, when attempted to escape. To more objectively quantify this difference, we implemented SHIRPA protocol designed for comprehensive phenotype assessment [15]. Semi-quantitative grip-strength assessment implied a diminished muscular tone of rpS6^P−/−^ mice (Fig. 1A). This inferiority was corroborated by a wire-maneuver test that showed that rpS6^P−/−^ mice lagged behind rpS6^P+/+^ mice when attempted to elevate their hind limbs to a horizontal wire (Fig. 1B). Next we observed that the ability of rpS6^P−/−^ mice to withstand a prolonged effort was severely impaired, as exemplified by significantly shorter time spent on the Rota-Rod (Fig. 1C). However, since poor performance in this test might reflect a defect in sensorimotor coordination, rather than muscle weakness, we examined the mice on a rodent treadmill. Fig. 1D demonstrates that the endurance of the knockin mice is profoundly diminished, relative to their wild-type counterparts. Taken together, these results attest to the role of rpS6 phosphorylation in normal muscle function.

### Muscle fiber growth relies on rpS6 phosphorylation

**Figure 2:**
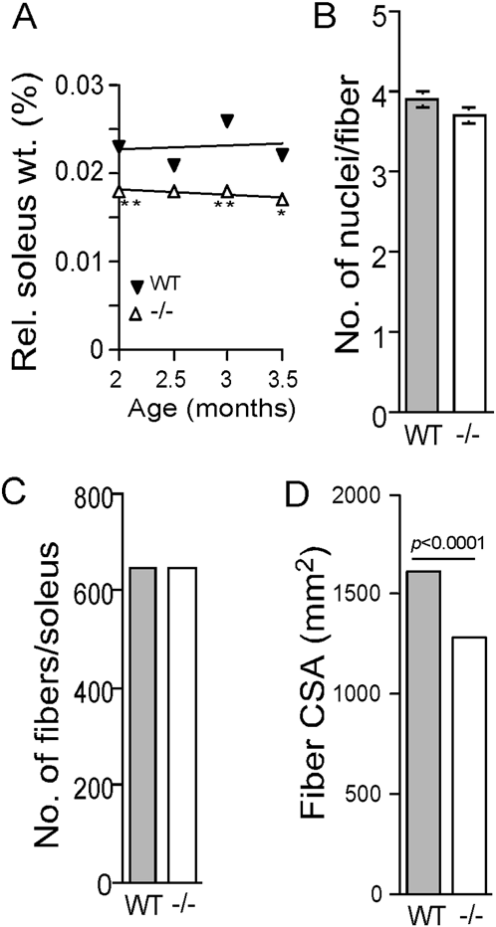
Soleus relative mass and CSA of its fibers are diminished in rpS6^P−/−^ mice. (A) The relative soleus weight from male mice was monitored at different ages and presented as the percentage of whole body weight. Each point represents mean±SEM, (bars are smaller than the symbol's size). * *P*<0.02, ** *P*<0.0001 versus rpS6^P+/+^ mice. (n = 4 to 52 soleus samples per each time point). (B) Number of myonuclei inside sarcolemma per fiber section. (C) Number of fibers comprising soleus muscles. (D) Cross-sectional area (CSA) of fibers. Muscles were excised from three 2-mo-old male mice for each genotype and immediately frozen. 5 µm-thick transverse cross-sections were collected along the entire length of the muscle at 100 µm intervals with a cryostat and stained with amylase/diastase in (B) in haematoxylin/eosin in (C) and reticulin in (D). Digital images obtained from the sections with the largest CSA were used for analysis. Numbers of fibers and nuclei per fiber were assessed by manual counting, and CSA was calculated from digital images. Values are presented as a mean±SEM. In (B) and (C) n = 3 and 4 age-matched male mice for each genotype, respectively. In (D) n = 1000 fibers from 3 age-matched male mice for each genotype. The SEM bars in (C) and (D) are invisible, because they are smaller than the width of the upper line in each column.

We have previously noticed that compromised glucose homeostasis in rpS6^P−/−^ mice is associated with smaller β-cell size and reduced content of pancreatic insulin [14]. Hence, we set out to examine the possibility that the apparent diminished muscle strength in the knockin mice might reflect a decrease in total muscle mass. Indeed, monitoring the total soleus weight in 2 to 3.5 month-old mice has demonstrated that the relative soleus weight is reduced by about 25% in rpS6^P−/−^ mice in comparison to that of rpS6^P+/+^ mice (Fig. 2A). Likewise, the weight of gastrocnemious muscles in 2-mo-old rpS6^P−/−^ male mice (n = 10) is 76% of that of their wild-type counterparts (74.5±3.6 [n = 10] and 98.3±4.2 [n = 9] mg, respectively). The optimal force of a muscle is largely determined by its physiological cross-sectional area (CSA) [16]. This latter variable reflects, among others, the CSA and/or number of individual fibers. Morphometrically, soleus fibers from rpS6^P−/−^ and rpS6^P+/+^ mice contain a similar number of myonuclei, testifying for the fusion of a similar number of myoblasts during fiber differentiation (Fig. 2B). Moreover, soleus muscles from both genotypes consist of a similar number of fibers (Fig. 2C). However, soleus muscle fibers of rpS6^P−/−^ show a 25% decrease in their CSA compared to that of rpS6^P+/+^ mice (Fig. 2D), quite similarly to findings in S6K-deficient mice [7]. It appears, therefore, that the decrease in the myofibers CSA can fully account for the diminished relative weight of the soleus in rpS6^P−/−^ mice, and this in turn might explain, at least partly the reduced muscle strength.

**Figure 3:**
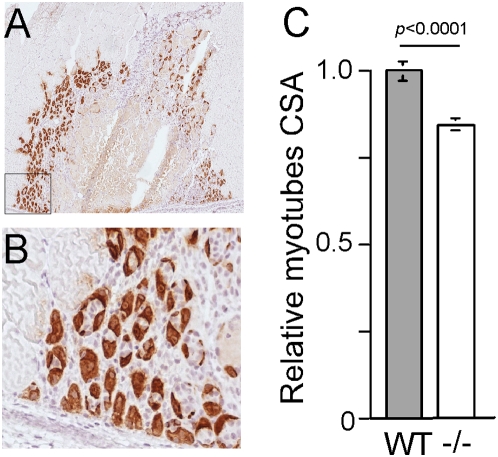
Newly formed myofibers are smaller in a rpS6^P−/−^ muscle. (A) Sections of tibialis anterior of 2-mo-old rpS6^P+/+^ and rpS6^P−/−^ mice were prepared 5 days after cryoinjury, and were immunostained for eMHC. One representative section is presented. (B) Enlargement of the framed area in (A). (C) Digital images of the immunostained sections were used for measurement of CSA of individual eMHC-positive myofibers, as described in “Experimental Procedures”. The average CSA of rpS6^P−/−^ myofibers (n = 327) was normalized to that of their wild type counterparts (n = 210), which was arbitrarily set at 1. The results are presented as average±SEM. *, p<0.0001.

The decrease in the CSA may stem from a smaller size of the fusing myoblasts and/or an impaired growth of the fully differentiated fiber. To monitor the size of newly formed myofibers, we exploited the ability of mitotically quiescent satellite cells in muscle to be activated in response to injury, giving rise to proliferating myoblasts. The latter undergo multiple rounds of division prior to terminal differentiation and fusion to form multinucleated myofibers [17]. The differentiating myoblasts express embryonic myosin heavy chain (eMHC) that serves as a specific marker of regenerating myofibers in the adult animal [18]. We have selected the tibialis anterior muscle, as it is a well-established model for muscle regeneration after cryoinjury through exposure to subfreezing temperature [19]. Sections obtained five days after the muscle had been injured, were subjected to immunostaining and morphometry (Fig. 3). Apparently, the average CSA of a newly formed rpS6^P−/−^ myofibers is 16% smaller than that of a wild-type counterpart. Due to their nearly round shape at this early stage of differentiation, the decrease in CSA can be translated into about 23% reduction in myofiber volume (Fig. 3C). It appears, therefore, that the diminished size of fibers is not confined to soleus. Moreover, the decline in muscle mass in rpS6^P−/−^ mice results, at least partly, from reduced size of the fusing myoblasts.

### The deficiency of rpS6 phosphorylation leads to a decrease in the abundance of contractile proteins

The fact that rpS6 is primarily a constituent of the ribosome has raised the possibility that its inability to undergo phosphorylation might affect (positively or negatively) the translation of a subset of mRNAs in the knockin mouse. In order to directly address this issue we carried out iTRAQ (Isobaric Tag for Relative and Absolute Quantitation)-based proteomic analysis of soleus from both wild type and rpS6^P−/−^ mice. The abundance of 16 out of 449 identified proteins decreased, whereas that of four proteins increased, by a factor of at least 1.5-fold (Table 1). It is conceivable that the decline in ten contractile proteins can partly explain the apparent muscle weakness. Interestingly, the abundance of both Apobec2 and carbonic anhydrase 3, two of the upregulated proteins, has previously been shown to increase under conditions of physiological or experimental muscle atrophy (aging or denervation, respectively) [20], [21].

**Table 1:** Proteins, whose abundance was up- or down-regulated in rpS6^P−/−^ soleus.

Protein description (Peptide No.)^a^	Gene Symbol	Protein Accession^b^	Fold Change
**Down-regulated proteins in rpS6^P−/−^ mice ** ***Contractile proteins***			
Myosin-8 (15)	Myh8	P13535	0.56
Myosin-1 (71)	Myh1	P12882	0.56
Myosin, heavy polypeptide 2, skeletal muscle, adult (33)	Myh2	Q9UKX2	0.59
Myosin-3 (3)	Myh3	P11055	0.60
Myosin light chain 1, skeletal muscle isoform (9)	Myl1	P05976	0.64
Myosin regulatory light chain 2, skeletal muscle isoform (17)	Mylpf	Q96A32	0.66
Isoform 1 of Tropomyosin beta chain (6)	Tpm2	P07951	0.50
Tropomyosin 4 (4)	Tpm4	P67936	0.61
Isoform 1 of Tropomyosin alpha-1 chain (16)	Tpm1	P09493	0.62
Troponin I, fast skeletal muscle (8)	Tnni2	P48788	0.58
***Mitochondrial proteins***			
Cytochrome b-c1 complex subunit 6 (4)	Uqcrh	P07919	0.63
NADH dehydrogenase [ubiquinone] iron-sulfur protein 4 (2)	Ndufs4	O43181	0.64
Cytochrome c oxidase subunit 3 (2)	mt-Co3	P00414	0.66
***Miscellaneous***			
Myoglobin (10)	Mb	P02144	0.63
Isoform 3 of LIM domain-binding protein 3 (11)	Ldb3	O75112	0.52
Hydroxysteroid dehydrogenase-like protein 2 (2)	Hsdl2	Q6YN16	0.59
**Up-regulated proteins in rpS6^P−/−^ mice**			
APOB mRNA editing enzyme catalytic polypeptide 2 (8)	Apobec2	Q9Y235	1.72
Ventricular myosin regulatory light chain (3)	Myl2	P10916	1.66
Carbonic anhydrase 3 (12)	CA3	P07451	1.66
Glycogenin-1 (3)	Gyg	P46976	1.53

Protein samples from soleus of wild type and rpS6^P−/−^ mice (2 of each genotype) were subjected to ITRAQ analysis, and the fold change in mutants relative to wild type was calculated, as described in Material and Methods. Only proteins, for which the difference between the measurements for the two samples of each genotype was ≤12%, were selected for this presentation.

a In parenthesis is the number of peptides used for identification of the protein and calculation of its relative abundance.

b Based on Protein Knowledgebase (http://www.uniprot.org/uniprot/).

### Muscles with unphosphorylatable rpS6 are deficient in readily available energy

**Figure 4:**
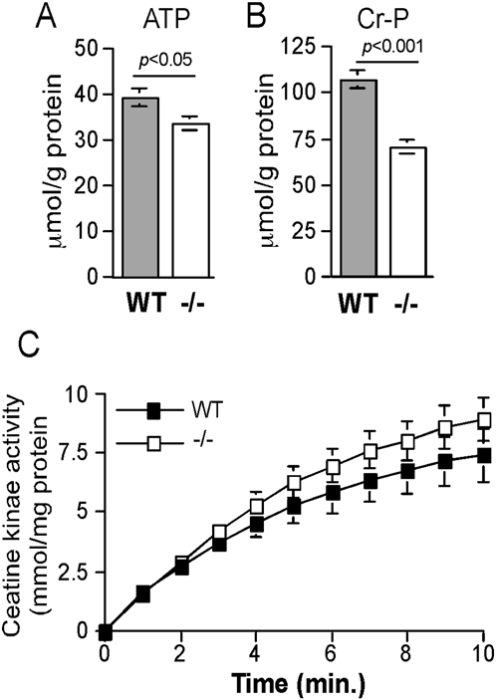
rpS6^P−/−^ muscles contain diminished amounts of ATP and phosphocreatine. ATP (A) and (B) PCr were determined in extracts from left and right hind-limb muscles. Results represent an average±SEM (n = 6 muscle samples for each genotype [3 aged-matched male mice each]). (C) The results for creatine kinase activity are presented as average±SEM of nmoles PCr produced at different time point normalized to protein content (n = 6 muscle samples for each genotype [3 age-matched male mice each]).

Contracting skeletal muscle exhausts first its free ATP and then utilizes the PCr/creatine kinase system as a means for regenerating ATP from ADP [22]. Hence, we assumed that the weakness displayed by rpS6^P−/−^ mice within a very short exercise time (in some cases within seconds), could have reflected diminished stores of ATP and/or PCr. Measurements of these variables have clearly demonstrated that this is indeed the case. Thus, ATP and PCr levels are lowered by 15% and 35%, respectively, in the hind limb of knockin mice, relative to wild type mice (Figs. 4A and 4B). Notably, the decreased content of PCr does not stem from impaired activity of creatine kinase (the difference in the kinetics between the two genotypes is statistically insignificant, Fig. 4C).

### Mitochondrial content and activity are similar in rpS6^P−/−^ and rpS6^P+/+^ muscle

**Figure 5:**
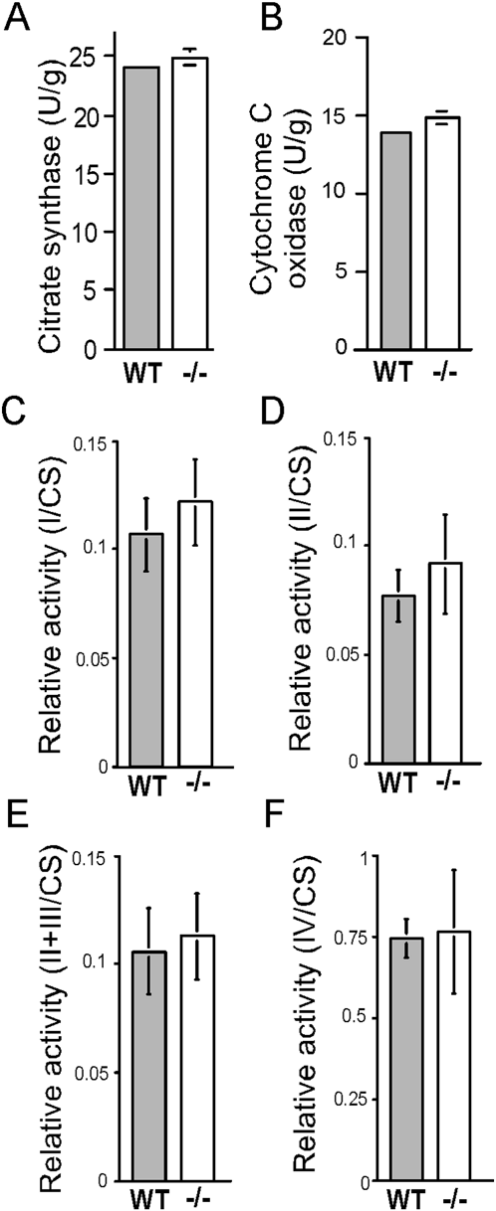
The amount of mitochondria and the activity of complexes of the oxidative phosphorylation system are unchanged in rpS6^P−/−^ muscle. Mitochondria were isolated from hind limb muscle of four age-matched rpS6^P+/+^ (WT) and rpS6^P−/−^ (−/−) male mice and were assayed for citrate synthase (A), cytochrome C oxidase (B), as well as complexes I (C), II (D), II+III (E) and IV (F) of the oxidative phosphorylation system. The activity of the different complexes was normalized to that of citrate synthase and the results are presented as relative activities. Vertical bars represent SEM (n = 4 age- matched male mice for each genotype).

To examine whether the reduced ATP content in rpS6^P−/−^ muscle may result from mitochondrial deficiency, we measured the activity of citrate synthase that is commonly used as an assessment of mitochondrial content [23]. The results show indistinguishable activity of this enzyme in muscles from both genotypes (Figs 5A). Likewise, the activities of cytochrome C oxidase and four complexes of the oxidative phosphorylation system were similar in mitochondria from rpS6^P−/−^ and rpS6^P+/+^ muscles (Figs 5B to 5F). Finally, measurements of oxygen consumption with glutamate and malate or succinate, as well as respiratory control ratio showed no significant difference between mitochondria from both genotypes (data not shown). These results suggest that the relative ATP deficiency in rpS6^P−/−^ muscle cannot be attributed to either a decline in mitochondrial mass or the activity of the measured parameters. Nevertheless, our proteomic analysis has demonstrated a decline in three mitochondrial proteins in the knockin soleus (Table 1) that might impair the mitochondrial function.

### Insulin induced signaling and glucose uptake is similar in muscles from wild type and rpS6^−/−^ mice

**Figure 6:**
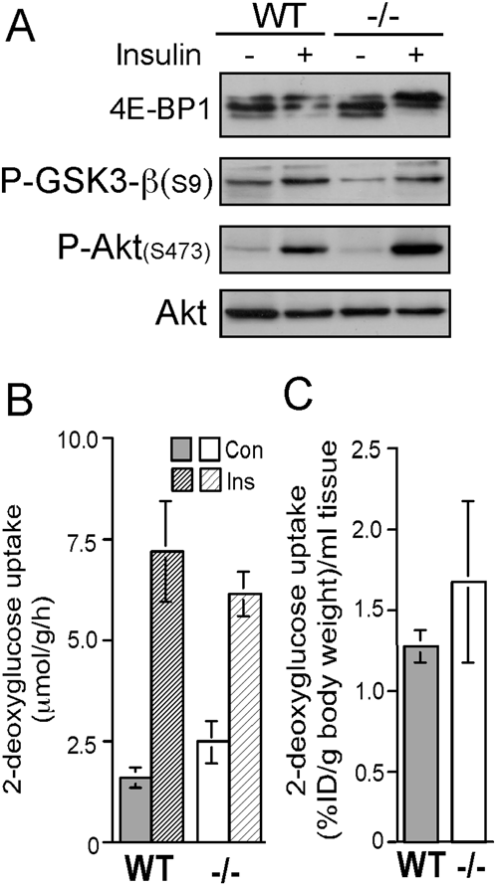
Insulin-induced signaling and glucose uptake are similar in rpS6^P−/−^ and rpS6^p+/+^ muscles. (A) Soleus muscles were excised from rpS6^P−/−^ (−/−) and rpS6^p+/+^ (WT) 2- to 3-mo-old male mice following 16 h starvation and intraperitoneal injection of saline (−) or 2.5 U of insulin/kg of body weight (+) for 5 min. Cytoplasmic extracts were subjected to Western blot analysis with the indicated antibodies. Note the enrichment for the upper band of 4E-BP1 (hyperphosphorylated form) upon insulin treatment. (B) Insulin-induced uptake of glucose into isolated muscle. Right and left soleus muscles were isolated from the hind limbs of 16 h starved rpS6^P−/−^ (−/−) and rpS6^p+/+^ (WT) male mice. 2-Deoxy glucose uptake is presented as µmol per gram tissue per h. The data are presented as average±SEM for 5 rpS6^P−/−^ and 4 S6^p+/+^ age-matched (7–9 weeks) male mice. (C) Mice were injected intraperitoneally with 0.25 U insulin/kg body weight and within seconds with 10–24 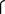
Ci [^18^F]fluoro-2-deoxyglucose (FDG) into the tail vein. The radioactivity concentration in hind limb muscles was estimated at 40 to 45 min after FDG injection by positron emission tomography. The concentration of radioactivity is presented as % injected dose (ID) per gm body weight per ml tissue. The data are shown as average±SEM for 5 male mice of each genotype.

Glucose tolerance tests have previously shown that rpS6^P−/−^ mice dispose an excess of blood glucose less efficiently than wild-type mice [14]. Glucose disposal is primarily carried out by the muscle and the adipose tissue through translocation of Glut4 transporter to the plasma membrane, in an Akt-dependent manner [24]. We have reasoned, therefore, that the relative deficiency in energy in rpS6^P−/−^ muscle might reflect impaired insulin signaling, and consequently diminished glucose uptake by this tissue. Initially we compared the ability of insulin to activate targets located downstream of Akt in soleus muscle. Fig. 6A shows that insulin induced phosphorylation of Akt at Ser473 and its activation, as exemplified by phosphorylation of Ser9 in GSK3-β, an Akt direct substrate, and a further downstream target, 4E-BP1 (a direct substrate of mammalian target of rapamycin [mTOR]). Notably, the stimulatory effect of insulin on Akt activity was at least as high in the rpS6^P−/−^ mouse as in its wild type counterpart. Next we monitored insulin-induced uptake of [^3^H]2-deoxyglucose, a non-metabolized glucose, by soleus and the results show no significant difference in this parameter between muscles from both genotypes (Fig. 6B). Likewise, measurements of insulin-induced uptake of [^18^F]2-deoxyglucose into leg muscles of a live animal, using positron emission tomography, demonstrated a comparable uptake by leg muscles of rpS6^P+/+^ and rpS6^P−/−^ mice (Fig. 6C). These results indicate that impaired insulin-induced signaling or glucose uptake cannot be considered as an explanation for the lower steady state levels of ATP or PCr in knockin mice.

### Stores of glycogen or triacylglycerol are not reduced in the rpS6^P−/−^ muscles

**Figure 7:**
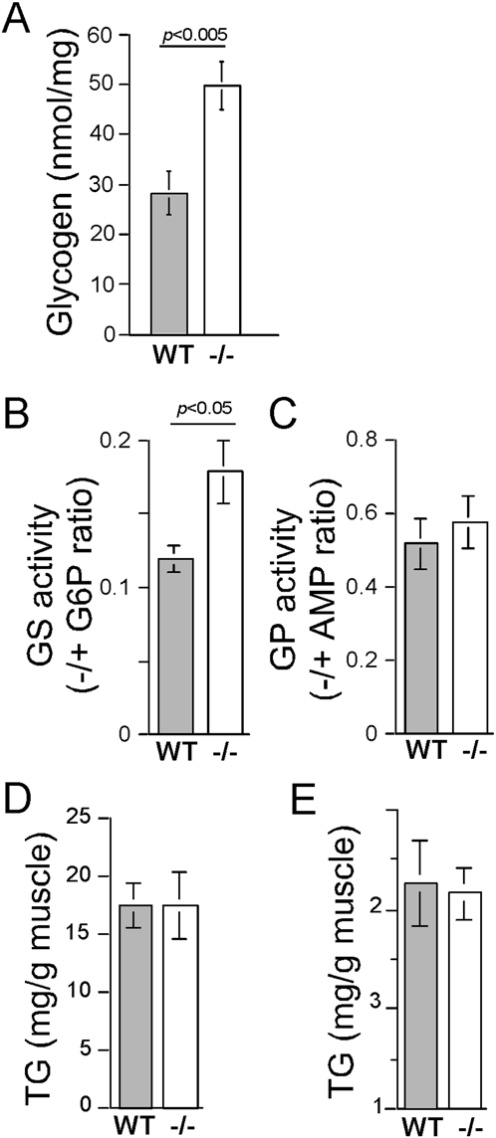
Stores of glycogen and triacylglycerol are larger and similar, respectively, in rpS6^P−/−^ muscle. (A) Soleus glycogen content. Soleus muscles were excised from 20 rpS6^P+/+^ (WT) and 15 age-matched rpS6^P−/−^ (−/−) male mice and their glycogen content was measured. (B) Glycogen synthase (GS) and (C) glycogen phosphorylase (GP) activities were assayed in the same extracts (n = 4 age-matched male mice for each genotype). (D) and (E) TG content in soleus and gastrocnemious, respectively. 5 soleus (left and right muscles were pooled) and 10 individual gastrocnemious muscles were excised from 5 rpS6^P+/+^ (WT) and 5 age-matched rpS6^P−/−^ (−/−) male mice and their TG content was measured. All results are presented as average±SEM.

Myofibers that undergo rapid exhaustion of ATP and PCr at early stages of sustained exercise initiate a number of metabolic pathways geared toward maintaining energy required for contraction [22]. Since, breakdown of glycogen and triacylglycerol (TG) are such metabolic means for rapid energy provision, we set out to compare the respective deposits in the muscle. Results presented in Fig. 7A show that not only was the glycogen storage not impaired in rpS6^P−/−^ muscle, it was nearly two-fold higher than that in the WT muscle. Moreover, this difference can be ascribed to increased glycogen synthase activity, rather than the impaired activity of glycogen phosphorylase (Figs. 7B and 7C) or the 25% decrease in total muscle mass (Fig. 2A), which was used for calculating glycogen concentration. Not surprisingly, therefore, the abundance of glycogenin-1, an essential protein for the formation of a glycogen granule, increased in rpS6^P−/−^ soleus (Table 1), as it is known to correlate with that of glycogen [25].

The TG content is similar either in soleus or gastrocnemious of both genotypes (Figs. 7D and E). Notably, the apparent difference in the relative content of TG in the two examined muscles seems to reflect their fiber types, as previous studies have shown that TG content is several fold higher in type 1 fiber-enriched slow twitch muscles (soleus) than type 2 fiber-enriched fast twitch muscles (gastrocnemious) [12]. Collectively, our biochemical analyses have disproved a role of impaired insulin-dependent glucose uptake, reduced mitochondrial content, diminished activity of oxidative phosphorylation complexes, as well as decreased glycogen or TG stores in the apparent deficiency of ATP and PCr in rpS6^P−/−^ muscle. However, we are currently unable to rule out downregulation of glycolytic flux, fatty acid oxidation or biosynthesis of either adenine nucleotides or creatine, as the reason for this deficiency.

## Discussion

The present study suggests two mechanisms to account for the compromised muscle strength in rpS6^P−/−^ mice: a decrease in total muscle mass, reflecting diminished CSA of myofibers, and reduction in readily available energy. Histological analyses suggest that the diminution in the myofibers CSA results from fusion of smaller size myoblasts, rather than a reduced number of fusing myoblasts during myotube differentiation. Interestingly, the association between the muscle weakness on the one hand, and the decrease in both the CSA of individual muscle fiber, as well as total muscle mass, is reminiscent of the phenotype of a mouse model for distal myopathy with rimmed vacuoles (DMRV), a human congenital disease, also known as hereditary inclusion body myopathy (hIBM) [26]. The smaller size of rpS6^P−/−^ fiber is in accord with our previous findings that rpS6 phosphorylation is involved in growth control of MEFs and β-cells [14]. Furthermore, it concords with the notion that the mammalian target of rapamycin complex 1 (mTORC1) functions as an integrator of cell growth signals (reviewed in [27]) through its downstream targets, S6K1 and eukaryotic initiation factor 4E-binding protein 1 (4E-BP1) [7], [28]. Thus, overexpression of S6K1 resulted in increased cell size that reflects an enhanced cell growth, rather than a delay in cell cycle progression [28]. Accordingly, the small size phenotype of S6K1^−/−^ myoblasts and rpS6^P−/−^ MEFs appears to result primarily from a defect in their growth [7], [14].

Many of the phenotypic manifestations of rpS6 knockin mice are similar to those observed in S6K1 knockout mice. These include: smaller size of β-cells, diminished insulin content in pancreas, hypoinsulinemia, glucose intolerance [6], [14], as well as smaller CSA of myofibers and reduced content of muscle PCr [[7], [10] and this work]. It is tempting, therefore, to assume that it is the failure to phosphorylate rpS6 that can account for these symptoms in both genotypes. However, the small size of S6K1^−/−^ myotubes is apparent, even though their rpS6 is still phosphorylated, most probably by S6K2 [7]. Moreover, several lines of evidence presented in this report suggest that, unlike S6K1^−/−^ muscle, the mechanism underlying the growth defect in rpS6^P−/−^ muscle does not involve AMPK activation, despite the reduced ATP content (Fig. 4A): a) Phosphorylation of acetyl-CoA carboxylase by AMPK lowers its activity and thereby the synthesis of its product, malonyl-CoA, thus relieving the inhibition of fatty acids uptake into mitochondria and thereby enabling their oxidation [29]. This is indeed reflected in diminished lipid content in S6K1-deficient muscle [10], but not in rpS6^P−/−^ muscles, whose TG stores were similar to that of wild type muscles (Figs. 7D and 7E). b) AMPK is known to inhibit skeletal glycogen synthase, through its phosphorylation [30], yet both glycogen synthase activity and glycogen storage are elevated in soleus of rpS6^P−/−^ mice (Figs. 7A and 7B). c) One of the mechanisms by which AMPK induces ATP production is by upregulating mitochondrial biogenesis [31], as evident for S6K1-deficient muscle [10], but not for rpS6^P−/−^ muscle (Fig. 5). d) AMPK is known to stimulate glucose uptake in skeletal muscle [29], yet our *in vitro* and *in vivo* glucose uptake measurements have failed to detect upregulation of basal or insulin-dependent glucose uptake in rpS6^P−/−^ muscle (Fig. 6). e) Assessment of AMPK activity through monitoring the phosphorylation of its direct substrate, acetyl CoA carboxylase, has revealed that neither its basal nor its induced activity following treatment of mice with AICAR, a prototypical AMPK activator, is higher in rpS6^P−/−^ soleus (data not shown). Taken together, our results indicate that rpS6^P−/−^ deficiency evokes a defect in muscular growth through a mechanism that deviates from that proposed for S6K1 deficient mice.

The distinction between the mechanisms operated by these two related deficiencies suggests that S6K1 regulates cell size predominantly through one or more of its multiple substrates [3], rather than rpS6. Indeed, SKAR, another S6K1 substrate, has been implicated in regulation of cell growth, as knockdown of its expression reduced the cell size [32]. Furthermore, inhibition of both S6K1 and S6K2 by the mTOR-specific inhibitor, rapamycin, is not sufficient to achieve a complete dephosphorylation of rpS6, as it is still phosphorylated at two of its five phosphorylatable serine residues by RSK [13]. Hence, rpS6^P−/−^ recapitulates a combined deficiency of both S6Ks and RSKs, or simultaneous inhibition of the mTOR-S6K and extracellular signal-regulated kinase (ERK)-RSK pathways. Conceivably, a different mechanism is predominately operative under such extreme conditions.

Finally, this study evokes an intriguing question regarding the mechanism(s) underlying the role of rpS6 phosphorylation in regulating processes as diverse as cell growth and ATP/PCr production, particularly in the face of the fact that rpS6 is primarily a structural protein of the ribosome. Several explanations can be proposed to account for these unique physiological functions of rpS6 phosphorylation: (a) The phosphorylation of rpS6 within, or outside, the ribosome affects the translation efficiency of specific mRNAs, as likely to be the case for the 20 soleus proteins identified here (Table 1). (b) rpS6 might be one of the many bifunctional ribosomal proteins, that can carry out extraribosomal tasks often unrelated to the mechanics of protein synthesis [33]. (c) Phosphorylated rpS6 might not affect protein synthesis, but instead interacts with cellular protein(s), which consequently becomes active or inactive, and thus affects the cell physiology. Indeed several extraribosomal proteins have been reported to be coimmunoprecipitated with rpS6, suggesting an *in vivo* interaction, either directly or indirectly with these proteins. [34], [35].

## Materials and Methods

### Animals

rpS6^P−/−^ mice, previously generated by homologous recombination [14], were genotyped as described [14] and kept, similarly to their wild-type counterparts in a hybrid background derived from the 129Sv and ICR mouse strains. Animals were maintained on a 12-h light/dark cycle and allowed free access to food. Animal experiments were carried out in compliance with the Hebrew University guidelines.

### Assessment of muscle strength

Three tests from the SHIRPA protocol [15] were used to assess abnormal phenotypes in the knockin mouse: (a) Positional passivity test (grip strength) provides assessment of the struggle response to sequential handling and might indicate a defect in muscle or lower motor neuron function. (b) Wire maneuver - Mice were held above a horizontal wire (1.5 mm diameter) by tail suspension and lowered to allow the forelimbs to grip the wire. Grip strength was assessed by measuring the time it took a mouse to raise its hind limbs and grip the wire. (c) Rota-rod test is designed to assess the mouse sensorimotor coordination and defects in motor function. Mice were placed on a 3 cm-diameter cylinder of an accelerating rota-rod (Rota-rod Treadmill 7650, Ugo Basile, Middlesex, UK) apparatus. This cylinder was gradually accelerated from an initial speed of 4 rpm to a maximum of 40 rpm, and the duration of time during which the mouse is able to remain on the rotating rod was recorded. The fourth test was based on treadmill performance for assessment of physical endurance. Mice were initially familiarized with the exercise protocol by walking or running on a rodent treadmill (built in the workshop of Technion-Israel Institute of Technology) on consecutive days, during which they performed 20 min of exercise with 0% grade. A day later mice were challenged with a speed of 20 m/min and 12.5% grade.

### Number of fibers and nuclei in soleus and cross sectional area of individual fibers

Soleus muscles of 7–9-week-old male mice were embedded in OCT (Optimal Cutting Temperature freezing medium; Sakura Finetec, Torrance CA), frozen in isopentane cooled on liquid nitrogen and stored at −70°C. Transverse 5 µm-thick cross-sections were collected along the entire length of the muscle at 100 µm intervals with a cryostat (Leica CM 1850). The sections with the largest CSA were used for analysis. Reticulin staining was used for the assessment of fiber size and number, whereas PAS (Periodic acid-Schiff)-Diastase staining was used to quantify the number of nuclei per fiber. Staining was performed utilizing Ventana Special Stains kits on Nexes Special Stains Module (Ventana Medical Systems Tucson, AZ). Digital images were obtained using Nikon Eclipse TE-2000E microscope equipped with Olympus DP70 camera. Image analysis for fiber CSA, number of fibers per muscle and number of nuclei per fiber was carried out using Image Pro Plus software (Media Cybernetics, Silver Spring, MD).

### The size of newly generated myoblast in adult mice

The tibialis anterior muscles were injured by applying a cold metal bar (5 millimeter spatula pre-cooled in liquid nitrogen) directly to the muscle for 10 seconds. This generated a cryoinjury in the muscle with a discrete border between uninjured and injured muscle, and this border remains clear and distinct during the regeneration of the injured tissue. The skin incision was stitched using silk sutures. Five days later, muscles were dissected, embedded in Tissue Tek O.C.T. compound and placed on a metal plate. The metal plate was then embedded into a bath filled with isopentane, which was placed in a larger bath with liquid nitrogen. The frozen muscles were kept in −70°C. Immunostaining of the cryo sections was performed using anti-embryonic myosin heavy chain (eMHC, a gift from Developmental Studies Hybridoma Bank, University of Iowa). Image analysis of the CSA of myofiber was carried out using Image-Pro Plus software that counts pixels within a close line that overlaps the border of individual myofibers.

### Protein Extraction and proteolysis

Tissues were homogenized in 8 M Urea containing 20 mM DTT and 400 mM ammonium bicarbonate. The samples (100 µg each) were reduced with 10 mM DTT (at 60°C for 30 min), modified with 100 mM iodoacetamide in 10 mM ammonium bicarbonate (room temperature for 30 min) and trypsinized in 10 mM ammonium bicarbonate containing trypsin [modified trypsin (Promega)] at a 1∶50 enzyme-to-substrate ratio, overnight at 37°C.

### Mass spectrometry analysis

The tryptic peptides were desalted using C18 tips (Harvard Inc.) dried and resuspended in 100 mM Hepes (pH 7.3). The iTRAQ™ Reagents were mixed with ethanol and each one of the reagents was transferred to one sample tube (30∶70 sample∶reagent ratio). Two mutant samples were labeled with 114 and 115 reagents and two wild type samples were labeled with 116 and 117 reagents. The tubes were incubated at room temperature for 1.5 h. All four iTRAQ™ Reagent-labeled samples were combined, cleaned on C18 and resuspended in 0.1% formic acid. 50 µg of the combined labeled peptides were separated by an on-line two-dimensional chromatography experiment (Multidimensional Protein Identification Technology). First the peptides were loaded on 15 mm of BioX-SCX column (LC Packing) and eluted with 10 salt steps of 0, 30, 60, 80,100, 120, 160, 200, 300, 500 mM ammonium formate in 5% acetonitrile and 0.1% formic acid, pH 3. The eluted peptides were further resolved by capillary reverse-phase chromatography (75 µ ID, 20 cm fused silica capillaries, J&W self-packed with 3 µ Reprosil-Aqua C_18_). The peptides were eluted with linear 85-minutes gradients of 5 to 45% and 15 minutes at 95% acetonitrile with 0.1% formic acid in water at flow rates of 0.25 µl/min. Mass spectrometry was performed by an ion-trap mass spectrometer (OrbitrapXL, Thermo) in a positive mode using repetitively full MS scan followed by collision induced dissociation (CID) and Higher Energy Collision Dissociation (HCD) of the 3 most dominant ions selected from the first MS scan. The mass spectrometry data was analyzed and compared using the Sequest software (Bioworks3.31, Thermo) searching the mouse section of the NR-NCBI database. Quantitative analysis was done using PepQuan (Thermo). The peak intensity of samples labeled with 115, 116 and 117 reagents was normalized to that of the sample that was labeled with the 114 reagent, which was arbitrarily set at 1. The relative level (or fold change) of a protein in the knockin mouse represents the ratio of the average value of the two mutant samples ([114+115]/2) to that of the two wild type samples ([116+117]/2).

### Measurements of ATP and phosphocreatine

Hind limb muscles (composed of gastrocnemious, soleus and plantaris) were rapidly removed and frozen in liquid nitrogen. Metabolites were extracted with perchloric acid and the levels of ATP, as well as phosphocreatine (PCr) were determined in neutralized supernatant by coupled enzyme assays [36], [37]. Metabolite levels were normalized to protein concentrations measured on parallel samples [38].

### Creatine kinase assay

The activity of creatine kinase was measured in extracts, freshly prepared from hind limb muscle by a coupled enzyme assay [39]. Enzyme activity was normalized to protein concentration.

### Western blot analysis

Immunoblotting was performed as described [40], using antibodies against rpS6, 4E-BP, Akt, phospho Akt (Ser473), phospho Akt (Thr308), (Cell Signaling Technology, Beverly, MA, USA). Exposures were chosen so that the chemiluminescent signals were within the linear response of the film and were quantified by ImageMaster VDS (Amersham Pharmacia Biotech).

### Mitochondrial analyses

Mitochondria were isolated from hind limb muscle by homogenization followed by differential centrifugation in sucrose [41]. The enzymatic activities of Citrate synthase (CS), rotenone sensitive NADH coenzyme Q reductase (complex I), succinate cytochrome C reductase (complex II+III), succinate dehydrogenase (complex II) and cytochrome C oxidase (complex IV) were determined by standard spectrophotometric assays [42]–[44], using a UVIKON XS spectrophotometer (SECOMAM, Ales France). Oxygen consumption with glutamate and malate or succinate in the presence of rotenone was performed on freshly isolated mitochondria using a Clark's type oxygen electrode (Hansatech, Norfolk UK). The respiratory control ratio (RCR) was calculated as the ratio of oxygen uptake in the presence and absence of ADP [41].

### In vitro muscle glucose uptake

Soleus muscles from the left hind leg were immediately isolated and incubated for 30 min at 30°C, in Krebs-Ringer bicarbonate buffer (KRB; 118.45 mM NaCl, 4.74 mM KCl, 1.17 mM MgSO_4_/7H_2_O, 1.27 mM CaCl_2_/2H_2_O, 1.18 mM KH_2_PO_4_, and 24.87 mM NaHCO_3_, pH 7.4) containing 2 mM sodium pyruvate. 100 nM of Regular Insulin (Eli Lilly) was added to identically treated contralateral muscles. Muscles were then rapidly transferred to KRB containing 1 mM 2-Deoxy [2,6-^3^H]glucose (1.5 µCi/ml; Amersham), and 7 mM [1-^14^C]D-Mannitol (0.5 mCi/ml; Sigma), and incubated for 10 min at 30°C. Insulin was present in the uptake media if present during preincubation. Both the preincubation and the uptake medium were continuously gassed with 95% O_2_-5% CO_2_. Subsequently, muscles were dried down briefly, frozen in liquid nitrogen, weighed upon thawing and incubated for 60 min at 65°C in 1 M NaOH. Following neutralization with 32% HCl, samples were centrifuged at 13 krpm for 10 min to precipitate particulates. Radioactivity was determined by liquid scintillation counting for dual labels, and the net uptake into the intracellular spaces was calculated after correction for the uptake into the extracellular space. [45].

### In vivo glucose uptake

Positron Emission Tomography (PET) with [^18^F]fluoro-2-deoxyglucose (FDG) was used to study the uptake of glucose in rpS6^P−/−^ and wild type mice. Prior to PET, the mice were given free access to food, and no restriction of movement. Mice were anaesthetized immediately prior to the study, and positioned in the scanner. Imaging was performed on a GE Discovery ST PET/CT. This is a clinical PET scanner, with 4-slice CT mounted on the same gantry for automated registration of the PET and CT scans. The PET scanner has a field of view of 50 cm diameter, 16 cm axially, and sensitivity of 8.6 kcps/kBq/cc (2D imaging mode), spatial resolution of 6.3 mm full width at half maximum (FWHM). A computed tomography (CT) scan was performed first to provide an anatomic image to assist with interpretation of the PET, and for use for attenuation correction of the PET image. Mice were first injected intraperitoneally with 0.25 U insulin/kg body-weight followed by an intravenous injection of 10–24 µCi of FDG into the tail vein. Dynamic PET imaging was started at the time of FDG injection, and continued for 45 minutes (10×30 s, 5×1 min, 7×5 min). PET data was corrected for scatter, random radioactive decay, and attenuation, and 128×128 pixel images were reconstructed of the central 25 cm of the transaxial field of view including the mice. Using CT images to assist in image interpretation, regions of interest corresponding to leg muscles were drawn on the PET images, and time activity curves were generated for these regions from the sequence of dynamic images.

### Glycogen determination

Glycogen was extracted from soleus samples by heating in 30% KOH, 5% Na_2_SO_4_ solution at 70°C for 15 min. Glycogen was precipitated by ethanol, and then subjected to 6N H_2_SO_4_ hydrolysis followed by neutralization with 625 mM NAOH and 55 mM Tris-HCl pH 8.0. The resulting concentration of free glycosyl residues was determined spectrophotometrically using a commercially available hexokinase-based assay kit (Teco Diagnostics, Anaheim, CA, USA).

#### Glycogen synthase activity assay

Glycogen synthase activity was determine as previously described [46] and is expressed as the ratio of glycogen synthase activity in the absence to that in the presence of 6.7 mM glucose 6-phosphate.

### Glycogen phosphorylase activity assay

Glycogen phosphorylase activity was determined as previously described [47] and is expressed as the ratio of glycogen phosphorylase activity in the absence (phosphorylase *a*) to that in the presence (total phosphorylase) of 3 mM 5′-AMP.

### Triglyceride determination

Gastrocnemious and soleus muscles were homogenized in 1 ml chloroform∶methanol (2∶1) per 100 mg tissue with Diax 100 homogenized (Heidolph, Germany) and lipids were extracted in 3 ml of the same mixture in tube rotator at 4°C. 0.04% CaCl_2_ was added (1/5 of the organic phase) 18 h later and the mixture was spun down at 2,800 rpm for 15 min. The organic (lower) phase was transferred to another tube and evaporated under N_2_ streaming. The triglyceride were dissolved in isopropanol and determined by monitoring the glycerol released using the combination of Free Glycerol and Triglyceride Reagents (Sigma, F6428 and T2449, respectively).
